# CTLA-4 correlates with immune and clinical characteristics of glioma

**DOI:** 10.1186/s12935-019-1085-6

**Published:** 2020-01-06

**Authors:** Fangkun Liu, Jing Huang, Xuming Liu, Quan Cheng, Chengke Luo, Zhixiong Liu

**Affiliations:** 1 Department of Neurosurgery, Xiangya Hospital, Central South University (CSU), 87 Xiangya Rd, Changsha, 410008 Hunan China; 2 Department of Psychiatry, The Second Xiangya Hospital, Central South University, Changsha, 410011 Hunan China; 3 Mental Health Institute of the Second Xiangya Hospital, Central South University, Chinese National Clinical Research Center on Mental Disorders (Xiangya), Chinese National Technology Institute on Mental Disorders, Hunan Key Laboratory of Psychiatry and Mental Health, Changsha, 410011 Hunan China; 4 Intensive Care Unit, Hunan Provincial Hospital of Traditional Chinese Medicine, Zhuzhou, China

**Keywords:** Immune checkpoint, CTLA-4, Immune response, Glioma, Prognosis

## Abstract

**Background:**

CTLA-4 is a well-studied immune checkpoint protein that negatively regulates T cell-mediated immune responses. However, the expression of CTLA-4 in glioma and the effects of CTLA-4 on prognosis in patients with glioma have not yet been examined.

**Methods:**

We investigated the protein level of CTLA-4 in human glioma samples, extracted genetic and clinical data from 1024 glioma patients to characterize CTLA-4 expression and its relationship with immune functions in gliomas. R language was used for statistical analysis.

**Results:**

Higher CTLA-4 expression was found in patients with higher grade, isocitrate dehydrogenase (IDH)-wild-type, and mesenchymal-molecular subtype gliomas than in patients with lower grade, IDH-mutant, and other molecular subtype gliomas. Further analysis showed that there was a strong positive correlation between CTLA-4 and the specific marker gene expression of immune cells, including CD8^+^ T cells, regulatory T cells, and macrophages in both databases, suggesting that higher CTLA-4 expression in the glioma microenvironment induced greater immune cell infiltration compared with that in gliomas with lower CTLA-4 expression. We further explored the associations between CTLA-4 and other immune-related molecules. Pearson correlation analysis showed that CTLA-4 was associated with PD-1, CD40, ICOS, CXCR3, CXCR6, CXCL12 and TIGIT. Patients with glioma with lower CTLA-4 expression exhibited significantly longer overall survival. Thus, these findings suggested that increased CTLA-4 expression conferred a worse outcome in glioma.

**Conclusions:**

In summary, our findings revealed the expression patterns and clinical characteristics of CTLA-4 in glioma and may be helpful for expanding our understanding of antitumor immunotherapy in gliomas.

## Background

Glioma is the most common primary malignant brain tumor in adults and is associated with a poor clinical course and prognosis. Despite improvements in surgical resection techniques, chemotherapy, and radiotherapy, the median overall survival (OS) of patients with glioma remains dismal. The discovery of the intracranial lymphatic system has challenged the classical dogma that the central nervous system (CNS) is immune-privileged and lacks immunosurveillance, which also supports the potential efficacy of immunotherapy in the treatment of glioma [1–4]. The recent striking clinical success of immune checkpoint inhibitors in other advanced cancers has encouraged the exploration of immune checkpoint blockade therapy in patients with glioma [5, 6]. A variety of clinical trials targeting programmed death (PD)-1/PD-ligand 1 (PD-L1), and cytotoxic T-lymphocyte antigen (CTLA)-4 have been investigated in patients with glioma to promote the robust antitumor T cell response [7].

As a well-studied immune checkpoint protein, CTLA-4 plays a crucial role in the tumor immunoreaction process. CTLA-4 is a major negative regulator of T-cell activation that interrupts costimulatory signaling and functions provided by cluster of differentiation (CD) 28:B7 binding [7]. Compared with the costimulatory molecule CD28, CTLA-4 has a 10- to 20-fold higher affinity to its ligands B7–1 (CD80) and B7–2 (CD86) [8]. By blocking the interaction between CTLA-4 and its ligands expressed by antigen presenting cells, inhibitors of CTLA-4 can block the inhibitory immune signal and restore anticancer immune responses. Monoclonal antibodies as CTLA-4 inhibitors were first reported in 1996 and have led to suppression of murine allergic contact dermatitis [9]. Inhibitors of CTLA-4 have also been shown to have remarkable success in clinical cancer immunotherapy in recent years. Ipilimumab is a fully humanized IgG1 subclass monoclonal antibody against CTLA-4 and was approved for melanoma therapy by the US Food and Drug Administration in 2011. Current trials are evaluating the use of this antibody for additional oncological indications. Another humanized anti-CTLA-4 antibody, tremelimumab, has been shown to elicit favorable responses in clinical trials against different tumor types. Combined with immune checkpoint inhibitors targeting PD-1/PD-L1, antibodies that block CTLA-4 may have applications as immunotherapies for the treatment of various malignancies, including glioma.

Several studies of immune checkpoint inhibitors targeting CTLA-4 have demonstrated promising benefits in patients with glioma [7]. The first large phase III trial of ipilimumab (a CTLA-4 inhibitor) plus nivolumab (a PD-1 inhibitor) in recurrent World Health Organization (WHO) grade IV glioma (glioblastoma) (NCT02017717) was initiated in 2014. With so many clinical trials, a comprehensive analysis of CTLA-4 expression will be required to identify the enrichment criteria of CTLA-4 in glioma clinical trials [10]. CTLA-4 was reported to highly expressed in high grade gliomas [11]. Doucette et al. [12] have analyzed the expression patterns of distinct glioma antigens and immune genes including CTLA-4 in different GBM subtypes using 544 samples from TCGA dataset. The mRNA level of CTLA-4 was higher in mesenchymal subtype compared with other GBM subtypes. However, more detailed and comprehensive reports of CTLA-4 expression in glioma is lacking.

Accordingly, in this study, we examined CTLA-4 expression in glioma specimens (WHO grade II-IV), explored the molecular and clinical characteristics of CTLA-4 in glioma by analyzing RNA-seq data from two databases (The Cancer Genome Atlas [TCGA] database and the Chinese Glioma Genome Atlas [CGGA] database). We expect that our findings will help facilitate the development of potential anti-CTLA-4 treatments in glioma.

## Methods

### Patients and samples

We used formalin-fixed and paraffin-embedded (FFPE) glioma specimens to detect the expression of CTLA-4. 58 glioma specimens from adult patients who underwent neurosurgical resection of gliomas at the Department of Neurosurgery, Xiangya Hospital were included in the analysis. We also included three additional normal brain tissues. Historical diagnosis of glioma was performed according to WHO classification. In total, 61 specimens were analyzed, including three normal brain tissues, 20 tumor specimens of glioma grade I-II, 12 tumor specimens of glioma grade III, and 26 tumor specimens of glioma grade IV. The study was approved by the ethics committee of the Xiangya Hospital, Central South University.

The RNA sequencing data and related clinical information were obtained from TCGA (http://cancergenome.nih.gov/) databases and CGGA (http://www.cgga.org.cn/) databases. 699 glioma samples (WHO grade II–IV) from TCGA database and 325 glioma samples (WHO grade II–IV) from CGGA database were included in our analysis. The IDH mutation data from TCGA were generated using whole exon sequencing or pyrosequencing. The IDH mutation data from CGGA were examined by pyrosequencing. Survival analysis in both databases were calculated from either the date of diagnosis or the start of treatment for a disease until death or last follow-up examination.

### Statistical analysis

Statistical analysis was mainly performed using R language (version 3.5.1; http://www.R-project.org) [13]. Several publicly available packages have been employed for figure generation. The heatmap was generated using the “pheatmap” package [14], Correlation between CTLA-4 and other checkpoint members were analyzed by Spearman correlation using the “circlize” package [15]. ROC curves were derived using the “pROC” package [16]. The Cox regression analysis was performed using the “survival” package [17], survival differences were compared by Kaplan–Meier method. All statistical tests were two-tailed and a p < 0.05 was considered to be significant in our study.

## Results

### CTLA-4 expression status in glioma

**Fig. 1:**
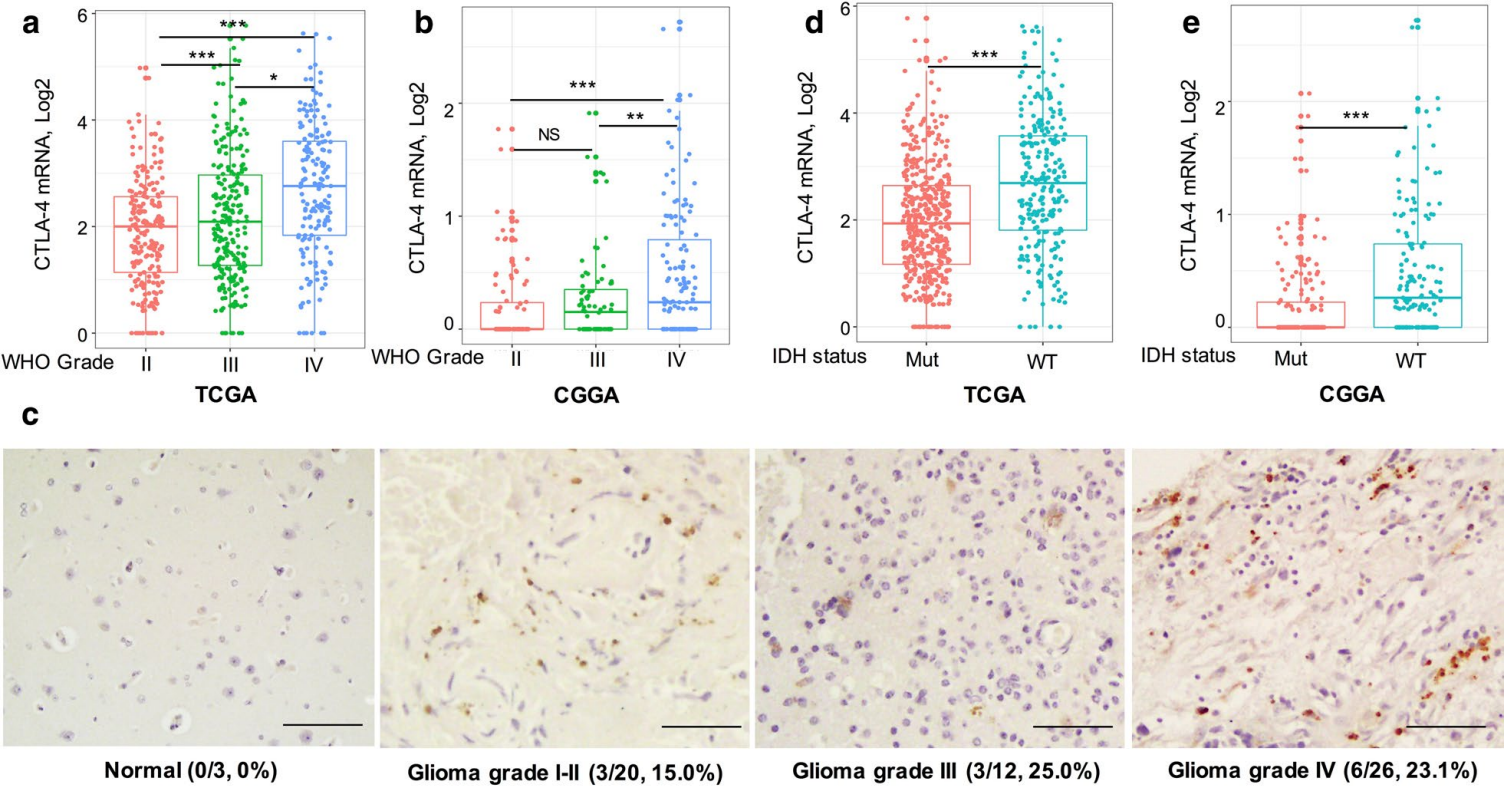
Relationship between CTLA-4 expression and clinical parameters of glioma. CTLA-4 expression in gliomas according to WHO grade status in the TCGA (**a**) and CGGA (**b**) databases. **c** Glioma tissues of different grades were immunostained for CTLA-4 using anti- CTLA-4 antibody and 3,30-diaminobenzidine (DAB; brown). Haematoxylin was used for nuclear counterstaining (blue). Numbers in parentheses indicate number and percentage of CTLA-4-positive samples out of number of total samples. Scale bars, 100 mm. CTLA-4 expression in gliomas according to IDH status in TCGA (**d**) and CGGA databases (**e**). * means p value < 0.05, ** means p value < 0.01, *** means p value < 0.001

We analyzed the expression of CTLA-4 in glioma according to WHO grade, IDH mutation status, and different subtypes in TCGA and CGGA databases. Gene expression profiling data in both databases were log-transformed for further analysis. In TCGA database, higher expression of CTLA-4 was detected in higher grade glioma samples. The highest CTLA-4 expression was found in WHO grade IV glioma (glioblastoma; Fig. 1a). We also validated our findings in CGGA database; similar to the results from TCGA database, higher expression of CTLA-4 was observed in grade IV glioma compared with that in grade II and grade III glioma (*p* < 0.01). The difference in CTLA-4 expression between grade II and grade III gliomas was not significant (Fig. 1b). These results indicated that CTLA-4 expression was positively correlated with pathogenic condition and malignancy in glioma. Further protein expression pattern of CTLA-4 in glioma also suggested that CTLA-4 expression was detected in high-grade glioma samples (Fig. 1c) (Additional file 1: Table S1).

Mutations in IDH are well-known molecular markers for the classification of different subtypes of gliomas [18]. Therefore, we examined CTLA-4 expression according to IDH mutation status in TCGA and CGGA databases. Gliomas possessing IDH mutations expressed lower CTLA-4 in TCGA database (Fig. 1d). Consistent with these findings, in CGGA database, CTLA-4 expression was significantly higher in IDH-wild-type gliomas than IDH-mutated gliomas (Fig. 1e).

**Fig. 2:**
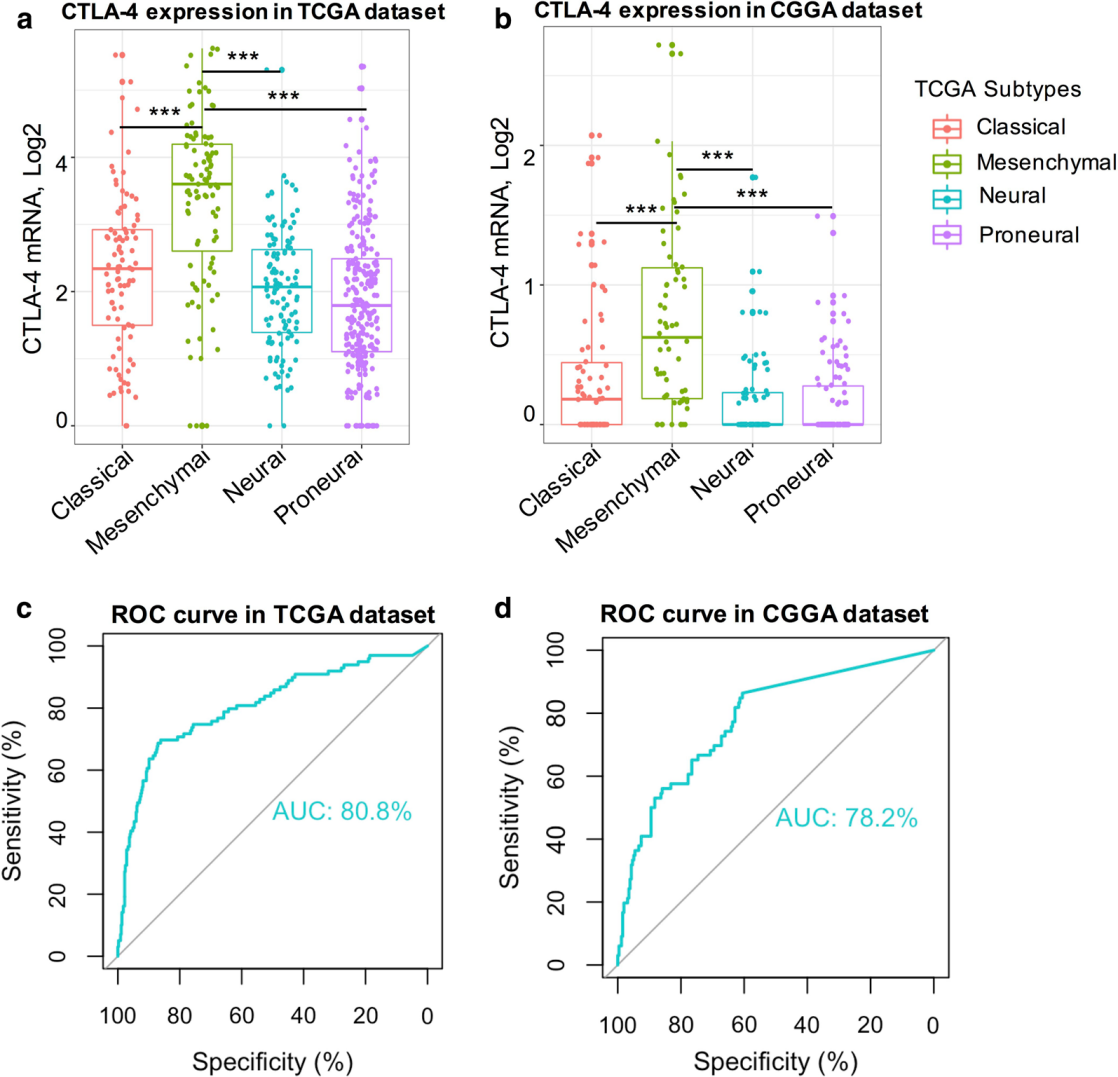
Relationship between CTLA-4 expression and glioma molecular subtypes. CTLA-4 expression pattern in different molecular subtypes of gliomas in TCGA database (**a**) and the predictive value analyzed by ROC curve analysis (**c**); CTLA-4 expression pattern in different molecular subtypes of gliomas in CGGA databases (**b**) and the predictive value analyzed by ROC curve analysis (**d**)

Different molecular subtypes of glioma are associated with different responses to treatment and prognoses [19]. To further characterize the relationship between CTLA-4 and TCGA-defined molecular subtypes, we measured CTLA-4 levels in different glioma subtypes. CTLA-4 expression in mesenchymal-molecular subtype glioma was significantly higher than in the other three subtypes in both databases (Fig. 2a, b). Receiver operating characteristic (ROC) curves were then generated using CTLA-4 expression and mesenchymal-molecular subtype to examine the predictive value in the databases we used. The areas under the curve were 80.8% and 78.2% for TCGA and CGGA databases, respectively (Fig. 2c, d). Our findings showed that CTLA-4 may serve as a good predictor for mesenchymal subtype glioma.

### Association between CTLA-4 expression and immune cell populations

**Fig. 3:**
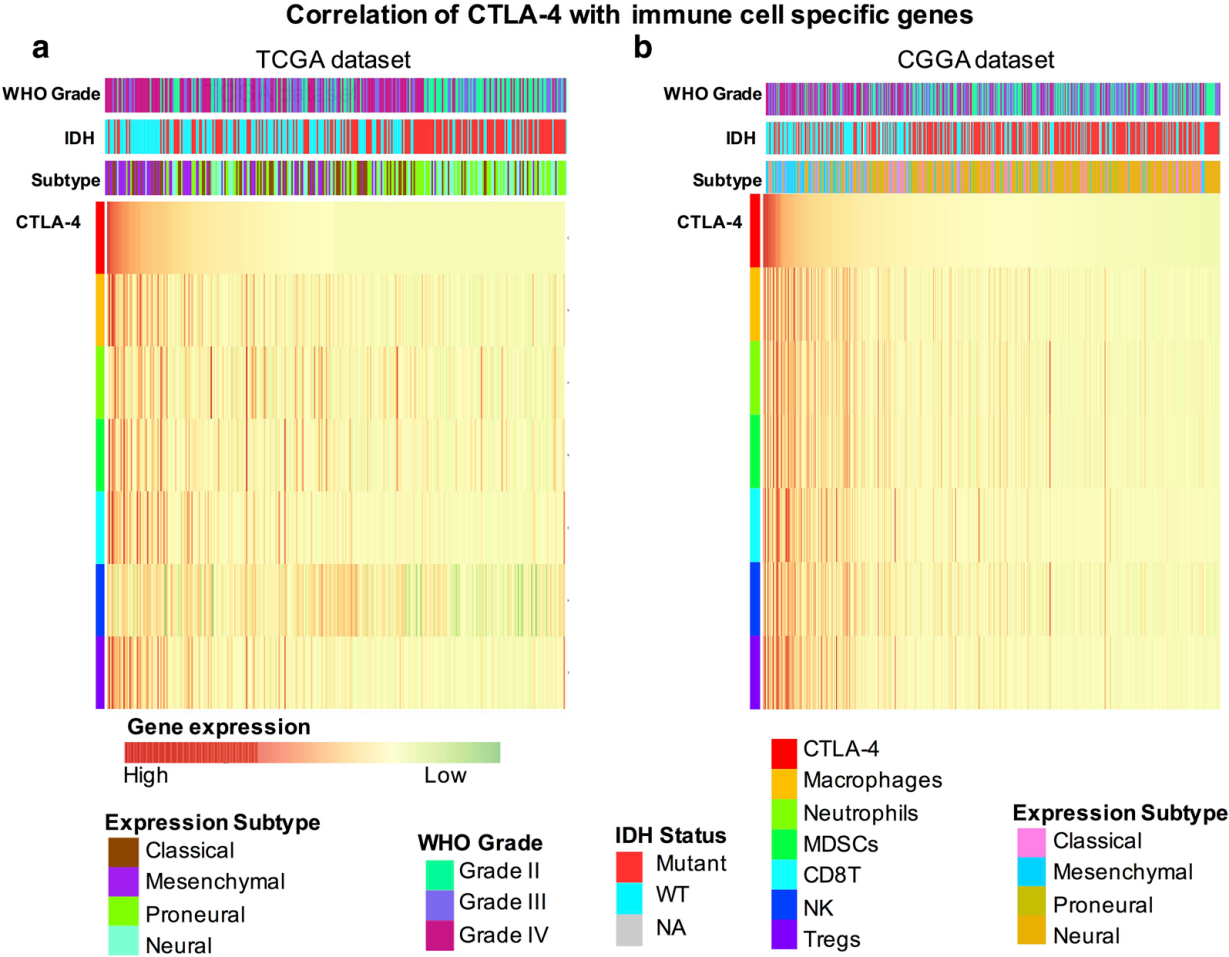
Heatmap analysis of the relationship between CTLA-4 and specific marker gene expression of all six immune cell types in TCGA (**a**) and CGGA (**b**) databases

Infiltration of immune cells plays a crucial role in immune and inflammatory responses against tumor cells [20]. As described by Etienne Becht et al. [21], we used the microenvironment cell populations-counter method to evaluated the association between CTLA-4 and immune cell populations in TCGA and CGGA databases. A detailed list of each immune cell type and related specific biomarkers is given in Additional file 2: Table S2. Six immune cells were analyzed, including CD8^+^ T cells, regulatory T cells (Tregs), natural killer cells, tumor-associated macrophages, myeloid-derived suppressor cells, and neutrophils. As shown in the generated heatmap, CTLA-4 was positively correlated with the specific marker gene expression of all six immune cell types examined, particularly in TCGA database (Fig. 3a, b). Detailed information regarding the r and *p* values between CTLA-4 and each immune cell type is given in Additional file 3: Table S3. The specific marker gene expression of all six immune cell types was significantly positively correlated with CTLA-4 expression in TCGA and CGGA databases. Moreover, there was a strong positive correlation between CTLA-4 and the following immune cells in both databases: CD8^+^ T cells (r = 0.65 in TCGA database, r = 0.62 in CGGA database), Tregs (r = 0.70 in TCGA database, r = 0.66 in CGGA database), and macrophages (r = 0.60 in TCGA database, r = 0.63 in CGGA database) (Additional file 4: Figure S1). These results indicated that higher CTLA-4 expression in the glioma microenvironment resulted in greater immune cell infiltration compared with glioma with lower CTLA-4 expression.

### Correlation between CTLA-4 and immune-related molecules

**Fig. 4:**
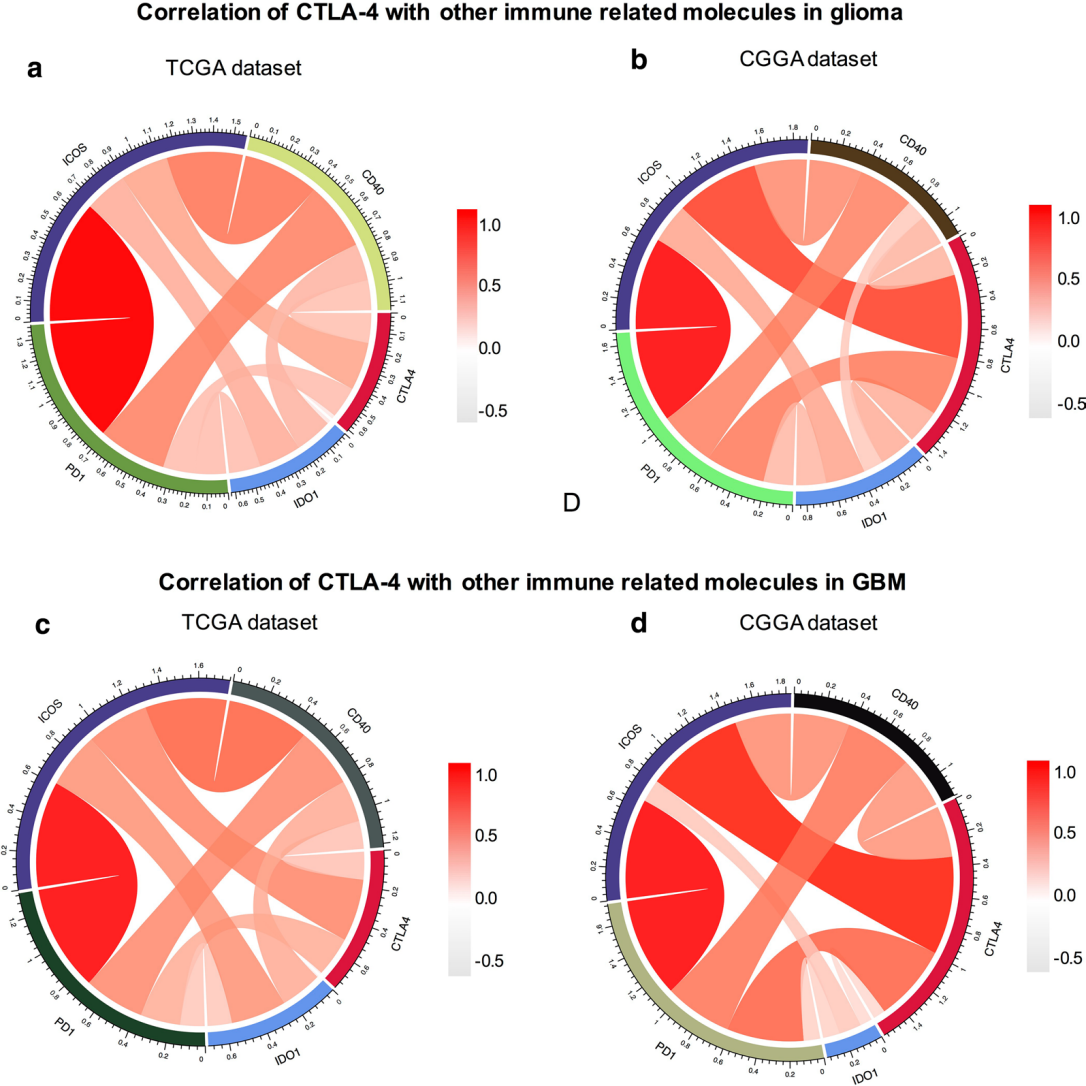
Association between CTLA-4 and immune related molecules in glioma. The correlations between CTLA-4 and PD-1, CD40, ICOS, IDO1 in glioma (**a**) and GBM (**c**) were analyzed based on TCGA database. The correlations between CTLA-4 and PD-1, CD40, ICOS, IDO1 in glioma (**b**) and GBM (**d**) were analyzed based on CGGA database

**Fig. 5:**
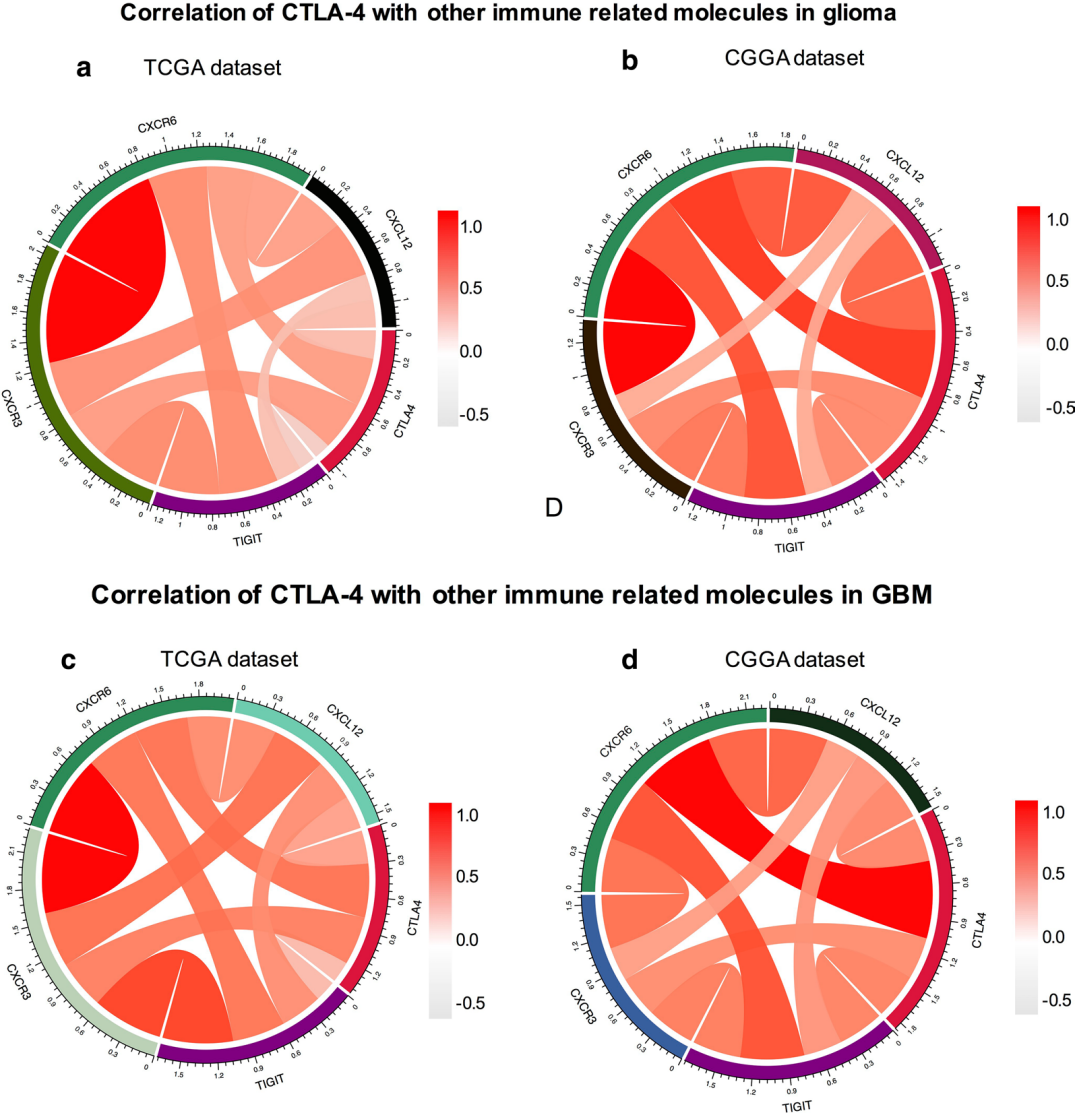
Association between CTLA-4 and immune related molecules in glioma. The correlations between CTLA-4 and CXCR3, CXCR6, CXCL12, TIGIT in glioma (**a**) and GBM (**c**) were analyzed based on TCGA database. The correlations between CTLA-4 and CXCR3, CXCR6, CXCL12, TIGIT in glioma (**b**) and GBM (**d**) were analyzed based on CGGA database

Combination therapies of different immune checkpoints inhibitors have shown great benefits compared with mono checkpoint therapy; indeed, combination therapies may yield higher anticancer immune responses and reduced immune-related adverse events [7, 22]. Researchers have shown that combination therapy with the CTLA-4 inhibitor nivolumab and the PD-1 inhibitor ipilimumab was more effective and resulted in significantly longer progression-free survival compared with monotherapy [23]. Therefore, we explored the correlation between CTLA-4 and other immune-related molecules. First, we examined the relationships between CTLA-4 and PD-1 [24], CD40 [25], indoleamine 2,3-dioxygenase 1 (IDO1) [26], and inducible T-cell costimulator (ICOS) [27], which have been reported in preclinical or clinical studies to be combined with CTLA-4 to enhance immunotherapy efficiency. Through Pearson correlation analysis, CTLA-4 was found to be significantly correlated with PD-1, CD40, and ICOS in TCGA and CGGA databases (Fig. 4a, b). CTLA-4 showed stronger associations with PD-1, CD40, and ICOS in patients with glioblastoma in both databases (Fig. 4c, d). Moreover, we analyzed the relationships between CTLA-4 and other immune-related molecules and showed that CTLA-4 was tightly associated with C-X-C motif chemokine receptor (CXCR) 3, CXCR6, and C-X-C motif chemokine ligand (CXCL) 12 in both TCGA and CGGA databases (Fig. 5a, b). Subsequent analysis showed that CTLA-4 was tightly associated with CXCR3, CXCR6, CXCL12, and T cell immunoreceptor with Ig and ITIM domains (TIGIT) in patients with glioblastoma (Fig. 5c, d).

### Prognostic value of CTLA-4 in glioma

**Fig. 6:**
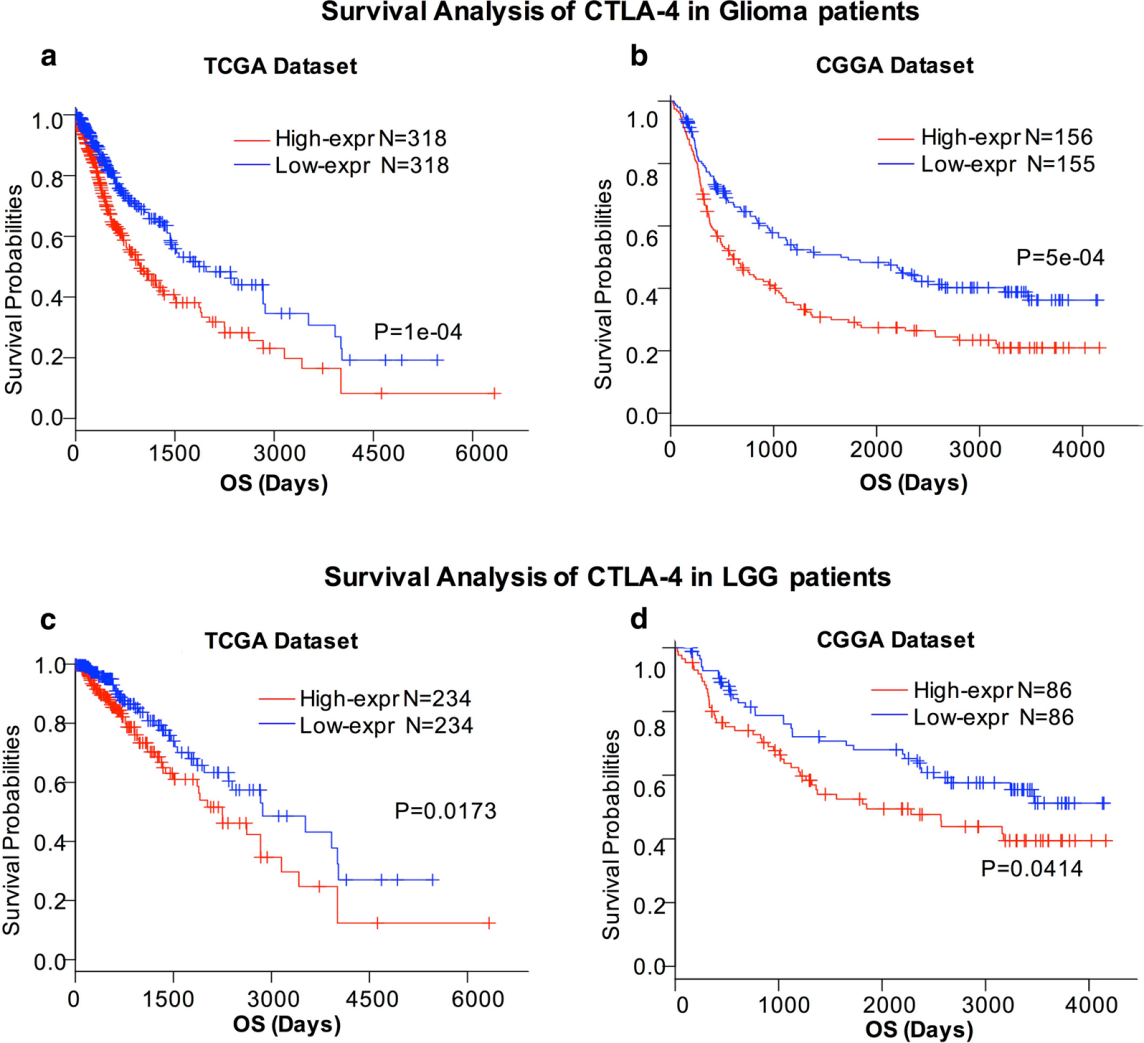
Survival analysis of glioma based on CTLA-4 expression. Higher CTLA-4 expression is associated with worse overall survival (OS) in patients with glioma and LGG based on TCGA database (**a**, **c**). Higher CTLA-4 expression is associated with worse overall survival (OS) in patients with glioma and LGG (**b**, **d**) in CGGA database

Based on our findings in TCGA and CGGA databases, higher expression of CTLA-4 was observed in higher grades of glioma, highlighting the possible relationship of CTLA-4 expression with poorer prognosis. Therefore, we divided patients with glioma into low- and high-expression groups to evaluate the prognostic value of CTLA-4. As shown in Fig. 6, patients with glioma with lower CTLA-4 expression exhibited significantly longer OS compared with patients with glioma with higher CTLA-4 expression in both TCGA and CGGA databases (Fig. 6a, b). Consequently, we also explored the prognostic value of CTLA-4 expression in patients with low-grade gliomas (LGGs). Similar Kaplan–Meier curves were observed in patients with LGGs (Fig. 6c, d). These findings indicated that increased CTLA-4 expression conferred worse outcomes in patients with glioma.

## Discussion

Immune checkpoint therapies targeting PD-1/PD-L1 and CTLA-4 have shown striking success against a variety of solid tumors. The genetic and clinical characteristics of PD-1/PD-L1 in gliomas have been explored, and the PD-1/PD-L1 axis has been shown to have malignant biological functions in glioma [28, 29]. Similar expression patterns for CTLA-4 were observed in our study. We found that CTLA-4 tended to be expressed at higher level in higher grade gliomas, IDH-wild-type gliomas, and mesenchymal-subtype gliomas. Similar expression patterns were also reported for other immune checkpoints such as PD-L1 [29], B7-H3 [26], TIM-3 [27], CD155 [30], and PTPN2 [28] in glioma. Besides, Kaffes et al. found highest immune cells presence in mesenchymal GBM compared with other subtypes [31]. These results suggested mesenchymal-subtype gliomas may be more amenable to immunotherapy. The expression pattern of CTLA-4 in our study can be a supplement to the existing findings.

Subsequent analyses of the relationships between CTLA-4 expression and immune cells showed that the specific marker gene expression of all six immune cell types was significantly positively correlated with CTLA-4 expression. Moreover, higher CTLA-4 expression was associated with worse prognosis in patients with gliomas.

CTLA-4 is expressed on activated conventional T cells and CD4^+^Foxp3^+^ Tregs and suppresses antigen-specific T-cell activation [7]. Previous studies have investigated CTLA-4 expression in patients with radically resected stage I–III non-small cell lung cancer (NSCLC) using tissue microarray immunohistochemistry. Higher CTLA-4 expression was found in nonsquamous NSCLC than in squamous NSCLC and in low Ki-67-expressing tumors [32]. CTLA-4 expression was also evaluated in CD34^+^ cells from patients with myelodysplastic syndrome, chronic myelomonocytic leukemia, and acute myeloid leukemia. Aberrant upregulation was observed in 8% of patients [33]. Another study showed that all melanoma cell lines analyzed expressed CTLA-4, and approximately 67% of melanoma specimens expressed CTLA-4 at different levels of intensity [34]. Other studies have examined the expression of CTLA-4 in nonlymphoid cells, suggesting that this molecule may be involved in more functions besides T cell response inactivation [35]. Fong et al. [36] analyzed the expression of CTLA-4 on peripheral blood lymphocyte subsets in GBM patients treated with DC vaccination and found CTLA-4 can predict survival in GBM patients. Higher expression of CTLA-4 was correlated with shorter survival after treatment. However, researchers also found blockade of immune-checkpoint inhibitors failed to recapitulate corresponding biomarkers-based clinical predictions using an orthotopic GL261-glioma mice model, which need further research to assess the applicability for clinical use [37].

Preclinical and clinical studies have demonstrated that combination of CTLA-4 and some immune-related molecules may enhance antitumor immunity. Saha et al. [38] showed that the triple combination of OHSV G47Δ expressing murine IL-12 with anti-CTLA-4 and anti-PD-1 antibodies extended survival with no pathological symptoms up to 9 months in a mouse glioma model. Field et al. [39] found blocking CTLA-4 priming with a whole cell vaccine eradicated tumor and prolonged survival in an orthotopic glioma model. Therefore, the relationships of CTLA-4 with PD-1, IDO1, CD40, and ICOS were evaluated, which have been reported to enhance immunotherapy efficiency in combination with CTLA-4. Blockade Inhibition of both PD-1 and CTLA-4 resulted in effectively restoration of T-cell proliferation and secretion of effector cytokines in tumors [40]. Additionally, clinical trials of anti-CTLA-4 (ipilimumab) and anti-PD-1 (nivolumab) have also been performed in patients with glioma [7]. The combination of anti-CTLA-4 monoclonal antibodies, anti-PD-L1 monoclonal antibodies, and the IDO inhibitor INCB23843 in the murine B16.SIY melanoma model showed markedly improved tumor control over single-drug treatment [26]. Moreover, a booster vaccination plus combinational treatment with CD40 stimulation and CTLA-4 inhibition resulted in complete tumor regression in a murine melanoma model [25]. ICOS belongs to the CD28/CTLA-4/B7 immunoglobulin superfamily and has been shown to play diverse roles in optimal antitumor responses mediated by anti-CTLA-4 therapy [27, 41]. In our study, we found that CTLA-4 was significantly correlated with PD-1, CD40, and ICOS in patients with glioma and glioblastoma. Recent studies have demonstrated that targeting the CXCR4/CXCL12 axis can restore sensitivity to CTLA4 and PD-1 checkpoints inhibitors [42]. Therefore, in this study, we analyzed the associations of CTLA-4 with CXCL12, CXCR3, CXCR6, and TIGIT, a new promising immune checkpoint-related protein [43]. We found that all of these molecules were tightly associated with CTLA-4 in patients with glioma. These results indicated the combination with these molecules can potentially enhance the efficacy of CTLA-4 blockade in cancer immunotherapy. Further researches can be performed to study the synergistic effect of these molecules.

## Conclusion

In summary, we investigated the associations of CTLA-4 expression with clinicopathological findings and IDH mutation status in gliomas. Moreover, we found that CTLA-4 was positively correlated with other immune-related proteins in glioma. Additional studies are needed to further explore the molecular mechanisms mediating CTLA-4 expression in gliomas and responses to anti-CTLA-4 therapy.

## Supplementary information

**Additional file 1: Table S1.** A summary of CTLA-4 expression prevalence determined with anti- CTLA-4 IHC assay.

**Additional file 2: Table S2.** The detailed list of each immune cell type and related specific biomarkers.

**Additional file 3: Table S3.** The detailed information of r value between CTLA-4 and each immune cell type.

**Additional file 4: Figure S1.** Correlation of CTLA-4 expression with immune cell-specific marker genes in TCGA (A) and CGGA (B) datasets. Each open circle represents a single patient with glioma. A regression line was fitted to the dot plot.

## Data Availability

The RNA sequencing data and related clinical information were obtained from TCGA (http://cancergenome.nih.gov/) databases and CGGA (http://www.cgga.org.cn/) databases. All other data and materials are available from the corresponding author upon reasonable request.

## Abbreviations

TCGA: The Cancer Genome Atlas
CGGA: Chinese Glioma Genome Atlas
IDH: isocitrate dehydrogenase
GBM: glioblastoma
GO: Gene Ontology
ROC: receiver operating characteristic curve
AUC: area under the curve
