# Supplementary material for: CTLA-4 correlates with immune and clinical characteristics of glioma
Source: Cancer Cell Int. 2020 Jan 6;20:7. doi: 10.1186/s12935-019-1085-6 (PMC6945521; doi:10.1186/s12935-019-1085-6)
Supplement: Supplementary file 1 — Additional file 1: Table S1. A summary of CTLA-4 expression prevalence determined with anti- CTLA-4 IHC assay. [file 12935_2019_1085_MOESM1_ESM.docx]

Table S1. A summary of CTLA-4 expression prevalence determined with anti- CTLA-4 IHC assay.

| Grade | *n* | Number of CTLA-4 positive | Percentage of CTLA-4 positive (%) |
| --- | --- | --- | --- |
| Normal | 3 | 0 | 0 |
| I-II | 20 | 3 | 15.0 |
| III | 12 | 3 | 25.0 |
| IV | 26 | 6 | 23.1 |
