# Supplementary material for: CTLA-4 correlates with immune and clinical characteristics of glioma
Source: Cancer Cell Int. 2020 Jan 6;20:7. doi: 10.1186/s12935-019-1085-6 (PMC6945521; doi:10.1186/s12935-019-1085-6)
Supplement: Supplementary file 2 — Additional file 2: Table S2. The detailed list of each immune cell type and related specific biomarkers. [file 12935_2019_1085_MOESM2_ESM.docx]

Table S2. The detailed list of each immune cell type and related specific biomarkers.

| Immune cell type | biomarker |
| --- | --- |
| Macrophages | CD14 |
| Macrophages | HLADRA |
| Macrophages | CD312 |
| Macrophages | CD115 |
| Macrophages | CD163 |
| Macrophages | CD204 |
| Macrophages | CD301 |
| Macrophages | CD206 |
| Neutrophils | CD11b |
| Neutrophils | CD16 |
| Neutrophils | CD66b |
| Neutrophils | ELANE |
| MDSCs | CD14 |
| MDSCs | CD16 |
| MDSCs | CD33 |
| MDSCs | ARG1 |
| CD8T | CD3E |
| CD8T | CD8A |
| NK | CD16 |
| NK | CD56 |
| Tregs | CD3E |
| Tregs | CD4 |
| Tregs | CD25 |
| Tregs | FOXP3 |
