# Supplementary material for: CTLA-4 correlates with immune and clinical characteristics of glioma
Source: Cancer Cell Int. 2020 Jan 6;20:7. doi: 10.1186/s12935-019-1085-6 (PMC6945521; doi:10.1186/s12935-019-1085-6)
Supplement: Supplementary file 3 — Additional file 3: Table S3. The detailed information of r value between CTLA-4 and each immune cell type. [file 12935_2019_1085_MOESM3_ESM.docx]

Table S3. The detailed information of r value between CTLA-4 and each immune cell type.

|  | TCGA |  | CGGA |  |
| --- | --- | --- | --- | --- |
| Immune cells | R | P-value | R | P-value |
| CD8T | 0.6539447 | <0.001 | 0.5094466 | <0.001 |
| Macrophage | 0.6023916 | <0.001 | 0.6349086 | <0.001 |
| MDSC | 0.5373754 | <0.001 | 0.4477182 | <0.001 |
| Neutrophil | 0.5345089 | <0.001 | 0.301696 | <0.001 |
| NK | 0.5335926 | <0.001 | 0.1982704 | <0.005 |
| Treg | 0.6989375 | <0.001 | 0.6567489 | <0.001 |
