# Supplementary figures and images for: CTLA-4 correlates with immune and clinical characteristics of glioma
Source: Cancer Cell Int. 2020 Jan 6;20:7. doi: 10.1186/s12935-019-1085-6 (PMC6945521; doi:10.1186/s12935-019-1085-6)

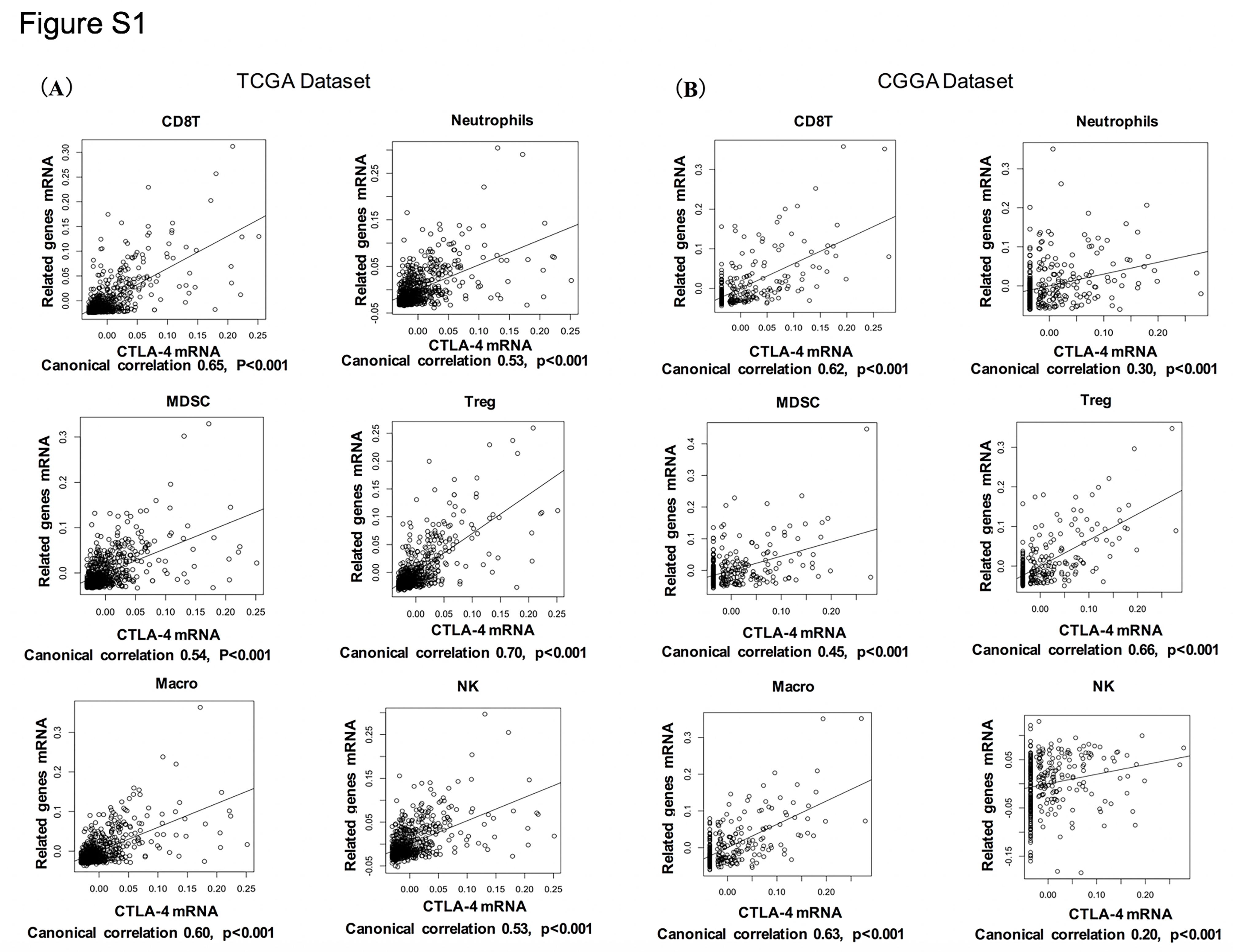

Supplement: Supplementary file 4 — Additional file 4: Figure S1. Correlation of CTLA-4 expression with immune cell-specific marker genes in TCGA (A) and CGGA (B) datasets. Each open circle represents a single patient with glioma. A regression line was fitted to the dot plot. [file 12935_2019_1085_MOESM4_ESM.jpg]
